# The Androgen Receptor in Breast Cancer

**DOI:** 10.3389/fendo.2018.00492

**Published:** 2018-08-28

**Authors:** Pia Giovannelli, Marzia Di Donato, Giovanni Galasso, Erika Di Zazzo, Antonio Bilancio, Antimo Migliaccio

**Affiliations:** Department of Precision Medicine University of Campania “L. Vanvitelli”, Naples, Italy

**Keywords:** breast cancer, triple negative breast cancer, androgen receptor, genomic and non-genomic actions, new therapies

## Abstract

Breast cancer (BC) is a hormone-related tumor. Despite the progress in BC therapy, this disease still remains the most common cancer amongst women around the world. This is likely due to the amazing BC heterogeneity. Accumulating evidence suggests a role for androgen signaling in BC. Nevertheless, a precise understanding of the mechanism of androgen action in this disease remains a challenging puzzle. Androgen receptor (AR) is often expressed in BC and several studies suggest that its role depends on the tumor microenvironment as well as the relative levels of circulating estrogens and androgens. However, the AR function in BC is still conflicting. Although AR expression is often associated with a favorable prognosis in EREstradiol Receptorα-positive (ERα +) BC, many findings suggest that, in some instances, high levels of AR can contribute to the therapy-resistance. Again, in ERα negative BC (ERα –), AR is mainly expressed in tumors with apocrine differentiation and a lower Nottingham grade. Moreover, AR stimulates cellular proliferation in triple negative breast cancer (ERα –, PgR –, and HER-2-Neu –). This finding is substantiated by the observation that high levels of circulating androgens are associated with an increased risk of developing BC in post-menopausal woman. Treatment of ERα- BC with AR antagonists, such as bicalutamide or enzalutamide, reduces, indeed, the tumor growth. In this review, we will analyze the putative role of AR in BC. Emerging therapies based on the use of new agonists or antagonists or inhibitors will be here discussed.

## Introduction

Breast cancer (BC) is the most common cancer and the fifth cause of death in women worldwide, with approximatively 1.7 million new cases diagnosed in 2012 [World Cancer Research Fund International https://www.wcrf.org; (1)]. The BC incidence is higher in industrialized countries, but the mortality is greater in less developed countries (1). This is much likely due to the easier accessibility, in rich countries, to medical care, from screening to advanced treatment, which makes possible to early detect the disease and to increase the possibility of recovery. The BC early detection remains a main issue in the fight against this cancer.

Despite the fact that in younger women exhibits more aggressive features and a worse prognosis, BC can be considered a prevalently post-menopausal disease (2). Factors as obesity, early age menarca, late age menopause and nulliparity have been shown to affect the development of post-menopausal BC, but the direct correlation is between genetic mutations and BC onset.

BC is a heterogeneous disease. Accordingly to the PAM50 classification, depending on the mRNA levels of ER, progesterone receptor (PgR), and human epidermal growth factor receptor 2 (HER2), we can recognize four different subtypes of BC that express at least one of these three receptors:

- Luminal type, which express ER and/or PgR, lack Her2: they can be further divided into A and B subtypes that differ for the expression of Ki67 (low levels in luminal A and high in B),
- Her-2 enriched BC, which are PgR negative, usually ER negative but express high Her2 mRNA levels and are characterized by a more aggressive phenotype and worse prognosis compared with luminal BC.
- Basal-like that are for the most part ER, PgR, and HER2 negative [triple negative, (3)] which are associated with the worst outcome among the BCs.

This heterogeneity explains why there is not an incisive therapy for BC treatment and highlights how it is important to develop new therapeutic strategies, alternative to the currently used drugs, such as Tamoxifen or Trastuzumab or Lapatinib, which are only useful when the target proteins (ER or Her2) are expressed. In this scenario, the androgen receptor (AR) is emerging as a new marker and a potential new therapeutic target in the treatment of BC (4).

Circulating androgens are detected at physiological conditions in women, and their levels change during life. Many studies have tried to understand the relationship between levels of androgens and breast cancer risk in women but the results are conflicting.

This paper is aimed to provide an overview of the role of androgen/AR axis in various BC subtypes, with particular focus on TNBC.

## AR: structure and function

**Figure 1:**
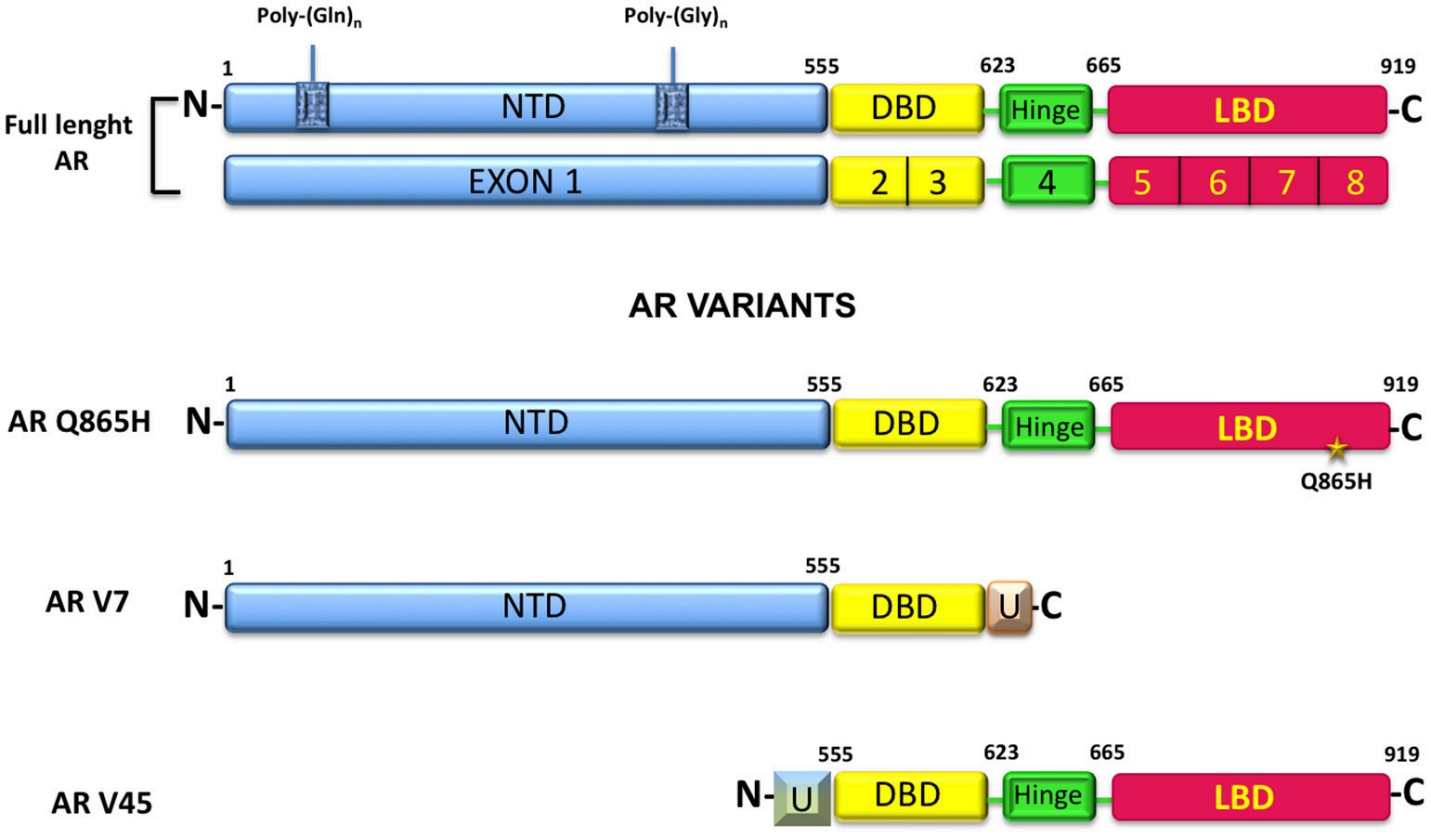
AR structure, alternative splicing variants and mutant commonly expressed in BC.

AR belongs to the steroid receptor superfamily and is classically considered a hormone-regulated transcription factor made up of 919-aminoacids encoded from a 180 kb gene located at the chromosome Xq11-12 (5). The receptor has three functional domains: (1) an amino-terminal domain (NTD, residues 1–555), (2) a DNA binding domain (DBD, resuidues 555–623), and (3) a carbossyl-terminal domain (residues 665–919). The hinge region (residues 623–665) connects the DBD- and the carbossyl-terminal domains (Figure 1). The amino-terminal domain is the largest domain and contains the activation function domain AF-1 that includes two separable transcription activation units, Tau-1 and Tau-5. The two Tau domains are required for the full activity of AR as well as the ligand-dependent interaction between the NTD and the LBD of the receptor. This interaction stabilizes the AR dimer complex and regulates the transcription of some AR-regulated genes (6). The amino-terminal domain includes a poly-glutamine (CAG) sequence with a variable number of ripetitions. The poli-Q lenght influences the folding and the structure of this domain and affects the AR-transcriptional activity, as minor lenght corresponds with a major AR activity (7, 8). The majority of the studies have found no relationship between the AR-CAG repeat lenght and BC risk, although Rebbeck & Co-workers have found that women carrying at least one AR allele with ≥28 CAG repeats were at significantly increased risk of breast cancer (9). AR also shows a GGC (polyglicine) repeats, but there is a limited number of studies concerning this polymorphism, with no evidence about its correlation with BC risk (10–12).

The AR-DNA binding domain is responsible for the binding to DNA that take place through the binding with a palindromic consensus sequence 5′-GGTACAnnnTGTTCT-3′ called androgen response element (ARE) and is only recognized by AR. Between the DBD and the hinge region, is localized the nuclear localization signal (NLS), responsible for AR nuclear import (6). The carbossyl-terminal domain (CTD) is the highly conserved domain between species and includes the activation function-2 (AF-2) and the ligand-binding domain (LBD), endowed with the androgen and anti-androgen binding sites (13).

Depending on the subtype, the wild type AR (AR wt) is expressed in 50–70% of BC. In MCF-7 cells and in T47D cells has been reported the expression of a membrane androgen receptor (mAR) and in estrogen receptor negative breast cancer MDA-MB 453 cells, showing a molecular apocrine differentiation, is expressed a mutated form of AR with a glutamine to histidine substitution, called Q865H (14). This mutant exhibits a reduced sensivity to 5α-dihydrotestosterone (DHT) and does not respond to non-androgenic ligands or AR antagonists (14). In BC circulating tumor cells (CTCs), an active splice variant of AR, AR-v7 is expressed. Expression of such mutant correlates with an increased number of bone metastases (15). Additionally, AR45 represents another splice variants expressed in MDA-MB231 and MDA-MB 453, together with the AR-v7. AR45 lacks of exon 1 and is preceeded by an N-terminal extension of 7-amino-acid long that inhibits the AR functions (16).

In sum, the presence of AR and/or its variants makes more complex the molecular scenario of BC.

## Androgens in BC

**Figure 2:**
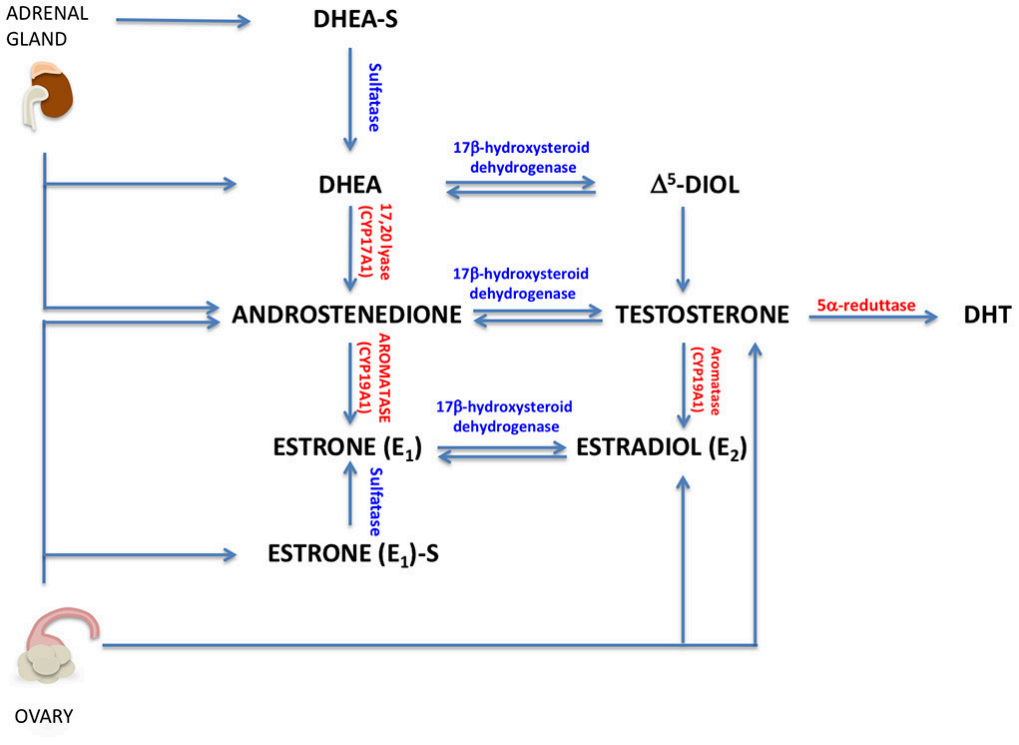
Circulating androgens in women. A brief representation of androgen and estrogen sinthesys in women (DHT, 5-α-dihydrotestosterone; Δ^5^-diol, androst-5-ene-3β, 17 β-diol).

To date, there is not a clear relationship between levels of circulating androgens and BC risk. In women, circulating androgens are dehydroepiandrosterone-sulfate (DHEAS), dehydro-androstenedione (DHEA), androstenedione (A4), testosterone, and DHT. DHEA, DHEAS, A4 are secreted by adrenal glands, while testosterone, DHEA and A4 are produced by ovaries [Figure 2; (17)]. Additionally, testosterone, DHT and their metabolites are produced in peripheral tissues, such as brain, bone and breast (18). All these hormones play key roles in reproductive system, muscle growth and prevention of bone loss.

Circulating androgens are detected in pre- and post-menopausal woman with different concentrations. Particularly, the levels of testosterone begin to decline in the mid-reproductive years, and the levels of adrenal androgenic steroids (adrostenedione and DHEA) decrease throughout post-menopausal life. Although the levels of androgens decline with menopause, this change, however, is less drastic than the decrease in circulating levels of estrogen and progesterone (19). This is mainly due to the reduced functionality of the ovaries that decreases the estrogen and progesterone production, but continues to synthesize constant levels of testosterone and, at lesser extent, androstenedione. A huge effort was made to establish a correlation between circulating androgens and BC risk. In pre-menopausal women, high levels of circulating testosterone increase the BC risk, but there are no data that demonstrate a link between high levels of others androgens and BC (17).

In post-menopausal women, high baseline serum testosterone is a strong prognostic factor for local relapse, controlateral BC, and distant metastases (20). Furthermore, high levels of others androgens (free testosterone, DHEAS, and androstenedione) and SHBG (steroid hormones binding globulin) are correlated with an increased post-menopausal BC risk (17).

However, not all the studies indicated a correlation between increased androgen levels and BC risk. Adly and Colleagues showed that the BC risk is linked only to higher serum levels of estrogens, independently of androgen levels. These hormones might only indirectly influence the BC risk, because of their conversion in estradiol (E2) by aromatase activity (21).

The variability of the presented data might be explained by the different techniques used to measure the testosterone levels in blood or *in situ* as well as the tribulations in interpreting data (22). To date, it is not clear if circulating androgens are a risk factor *per se* or as substrates for estrogens synthesis in breast tissues and BC. Maybe they can acts in both ways in all BC that express ER, but certainly not in ER-negative BC.

## The role of AR in BCs expressing ER

ER-positive BCs are classified in luminal A and luminal B subtypes. Both are ER+ and PgR+, but the luminal A are low proliferating BC. Luminal B are divided in two subtypes: HER2 negative subtypes that can express or not the PgR (the ER+/PgR+ have high proliferative property) and HER2 positive (ER+/PgR+ or PgR–/HER2+). AR is expressed up to 90% of ERα positive BC and its expression is related to a favorable prognosis. In this sub-group of tumors, AR seems to inhibit the cellular proliferation induced by estradiol and to have a favorable prognostic value. AR might modulate the ER signaling by interfering with both genomic and non-genomic actions.

AR is a transcription factor that binds specific AREs on DNA. In its inactive state, AR is located in cytoplasm and is bound to heat shock proteins (HSPs) 70 and 90. Upon androgen stimulation, AR is released by HSPs and translocates into the nucleus, where it recognizes and regulates androgen responsive genes. In ER-positive BC, AR could interfere with ER-dependent transcription by competing for the binding to the same sites or facilitating the ER binding to the DNA. In ER- and PgR-positive BC cells, AR signaling exerts inhibitory effects on cell growth (23). In these cells, ligand-bound AR moves into nucleus and binds to EREs, competing with ER and PgR. In PgR-negative BC cells, AR has a protumorigenic role and increases the ER gene transcription (24). By using the yeast and mammalian two-hybrid systems, Panet-Raymond and Colleagues have shown that AR and ERα directly interact through the C-terminal ERα ligand-binding domain and either the N-terminal AR transactivational domain or the full-length AR. This interaction can modify the transcriptional activity of both receptors with a stronger effect of AR on the ERα transactivation (25). In MCF-7 cells, the treatment with enzalutamide, an AR inhibitor, decrease by 50% the estradiol induced ER-binding sites on the chromatin highlighting the role of AR in ER-recruitment to DNA (26). The transcriptional interference between AR and ERα can be due also to shared coactivators. In MCF-7, ARA70, an AR coactivator, interacts with ERα and increases its transcriptional activity. This effect should be linked to the relative expression of AR and ERα; when AR is overexpressed (AR:ERα 5:1), ARA70 synergizes with AR and downregulates ERα transcriptional activity (27).

AR might also modulate the non-genomic actions regulated by ER (28). In breast cancer MCF-7 cells, about 8% of the total number of ERα and AR co-immunoprecipitates under basal conditions (29). Estradiol stimulation triggers the formation of a ternary complex between ER, AR and the cytoplasmic tyrosine kinase Src. Such complex rapidly and transiently activates the downstream pathway leading to MAPKs and cell proliferation (30). ER interacts with the SH2 domain of Src through the amino acid sequence surrounding phosphorylated Y537 within the C-terminal part of ERα and AR interacts with the SH3 domain of Src through a proline-rich domain. Disruption of the AR/Src interaction by a small, S1 peptide that mimics the AR poly-proline rich sequence, weakens the ER/Src complex assembly and inhibits the androgen induced MCF-7 proliferation (29, 31). Similar findings were observed in MCF-7 stimulated with epidermal growth factor (EGF), thus confirming that EGF signaling depends on the association of the ER and AR with Src in BC cells. In both cases (androgen or EGF stimulated MCF-7 cells), AR in association with ERα mediates the downstream events controlling cell cycle progression (32).

In MCF-7 cells, the treatment with the non-aromatizable androgen 5α-dihydrotestosterone (DHT) inhibits the cellular proliferation in different ways. It can reduce the cyclin D_1_ expression (33) and induce the association of AR and insulin growth factor 1 (IGF-1) thus increasing the AR stability and transcriptional activity (34). Stimulation of MCF-7 cells with Mibolerone (Mb), a synthetic androgen used to minimize the metabolic conversion of androgen to estrogenic compounds, reduces the cell proliferation rate by inhibiting the aromatase expression and activity and so the *in situ* estrogen production (35).

In ER-positive ZR75 and MCF-7 cells, activated AR up-regulates the ERβ levels [mRNA and protein; (36)]. This occurs through a recruitment of AR at ARE localized in ERβ promoter and lastly inhibits cancer cell growth, as ERβ prevalently acts as a tumor suppresson in BC (36). Again, treatment of MCF-7 cells with mibolerone (Mb) decreases cellular proliferation. This action is mediated by the binding of AR to an ARE sequence localized in the miR-21, which recruits the histone deacetylase 3 (HDAC3) and downregulates the miR-21 expression (37). This regulatory mechanism might represent a general example of AR genomic function, independent of ER status.

However, the findings reported above cannot allow concluding that AR exerts univocally a protective, anti-proliferative effect in ER+ BC. In fact, AR in concert with the lysine-specific demethylase 1 (LSD1) also induces the epithelial-to-mesenchymal transition (EMT) in BC cells in an ER alpha independent way. Upon DHT stimulation, AR and LSD1 interact to AREs localized at the promoters of E-cadherin and vimentin to regulate their expression, thus promoting metastasis formation (38).

In addition to classic AR, mediating transcriptional and non-transcriptional actions in BC, expression of mAR has been also reported in both MCF-7 and T47D (39, 40). This receptor is inhibited by pertussin toxin, but not anti-androgens, indicating that it can truly be a G-protein coupled receptor. To date, contribution of this receptor to BC progression and therapy escape are still under investigation.

Furthermore, some studies indicate that AR is responsible for BC chemoresistence (41, 42). In several specimens of Tamoxifen-resistant BC, low ER mRNA and high AR mRNA levels have been detected. Treatment with the antiandrogen Casodex reverses this resistance and indicates that AR signaling is directly involved (42). AR is also responsible for the aromatase inhibitor (AI) resistance in ER-positive BC cells (43). In ER-positive MCF-7 cells engineered to overexpress aromatase and AR (MCF-7 AR Arom cells) treated with androstenedione (AD), the nonsteroidal AI, anastrazole (Ana) does not inhibit growth and ER transcriptional activity. This effect could be due to an increase in pAKT and pIGF-1R levels as well as to a co-operation between AR and ER that might induce the up-regulation of AR and ER-responsive gene expression. AR antagonists, or the antiestrogen fulvestrant or the IGF-1R and AKT inhibitors restore the sensitivity to Ana (43).

In conclusion, whatever the mechanism, AR stimulates or inhibits cellular proliferation, promotes metastatization or resistence to therapies in ER-positive BC cells. These opposing actions could depend on the multitude of proteins interacting with AR.

## The role of AR in BCs not expressing ER

ER-negative BCs might express or not HER2 [ER–/PgR–/HER2+; ER–/PgR–/HER2–; (44)]. Recently, ER-negative BCs have been further classified for the expression of AR in triple negative (TNBC) or quadruple negative BC (AR–). There are different subtypes of AR+ TNBC (i.e., molecular apocrine and luminal AR–LAR-BC) in which the prognostic value of AR is not completely clear (45). AR acts both in a genomic and in a non-genomic way promoting, in most cases, the cellular proliferation.

Treatment of HER2+/ER–/AR+ BC cells (LAR-BC cells), MDA-MB453, with DHT stimulates cell growth by activating the oncogenic Wnt and the HER3 signaling pathway. In these cells, AR transcriptionally regulates the levels of Wnt7b, which in turn activates the Wnt pathway and stimulates the nuclear translocation of β-catenin that interacts with AR. In nuclei, the AR/β-catenin complex in concert with the transcription factor FOXA1 recognizes the regulatory regions of HER3 and increases its transcription thereby promoting cell growth (46). It has been also shown that AR regulates the Wnt/β-catenin pathway not only in LAR-BC cells but also in TNBC. They use two BC models, MDA-MB453 (HER2+/AR+) and MDA-MB231 (triple negative/AR+) cells and observe that DHT stimulation induces cellular proliferation. Bicalutamide, but not enzalutamide, inhibits this effect. The observed cell proliferation is likely due to an interaction between AR and β-catenin that regulates C-MYC, a downstream target of Wnt/β-catenin pathway (47). Again in TNBC MDA-MB231 and Hs578T cell lines, the DHT-induced cellular proliferation seems to be a consequence of the the G-protein coupled estrogen receptor (GPER) downregulation due to the binding of activated AR to GPER promoter. GPER is, indeed, largely expressed in TNBC and the androgen stimulatory effect on cell proliferation is inhibited by treatment with the GPER agonist, G1 (48). All these described effects are due to an exquisitely transcriptional mechanism elicited by AR.

**Figure 3:**
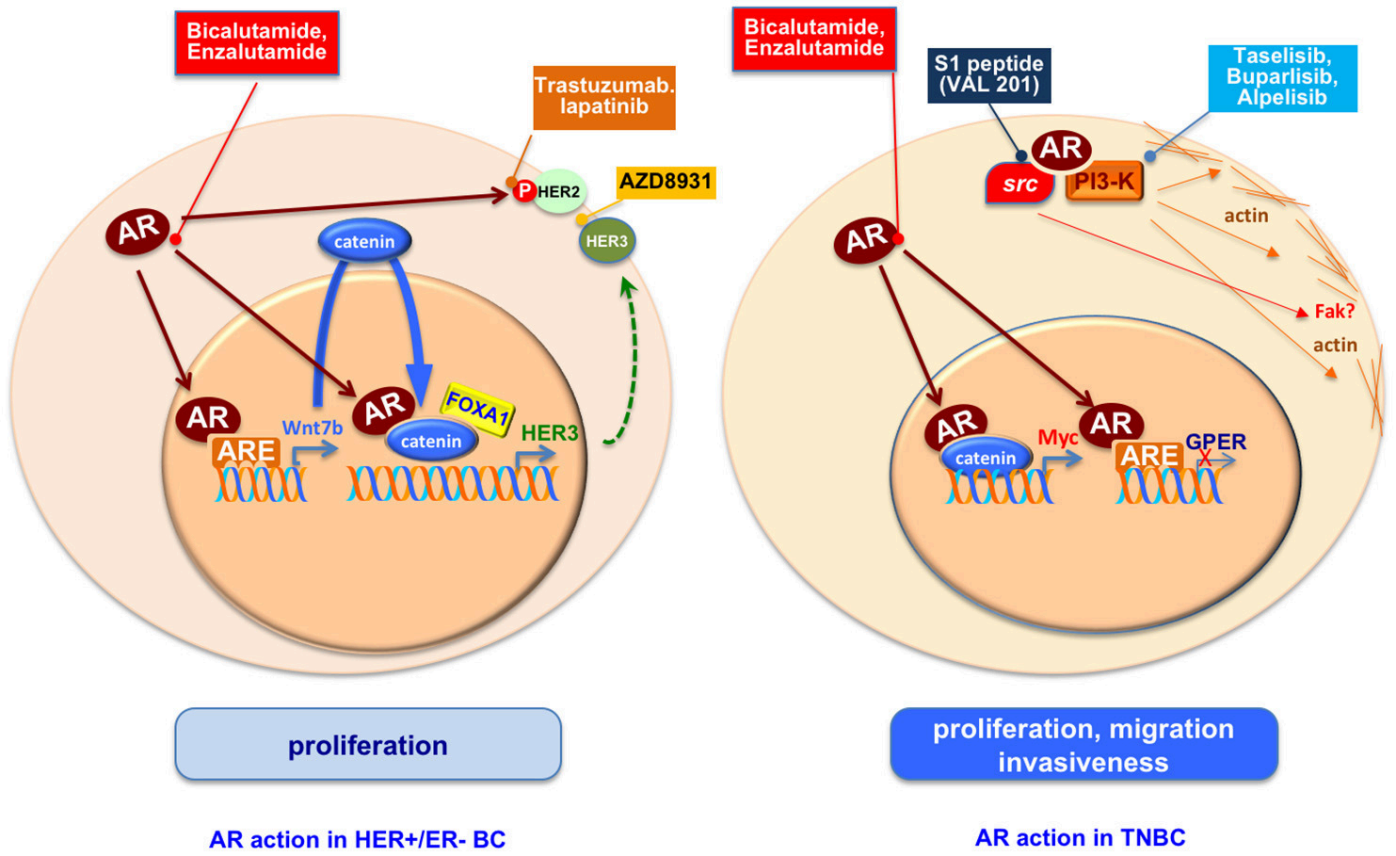
An overview of mechanisms activated by AR in HER+/ER– and TN-BC cells. AR regulates proliferation, migration and invasiveness in ER-BC through genomic and non-genomic pathways. The use of AR antagonists, inhibitors of AR activated proteins as HER2, HER3 or PI3K, or the S1 peptide (Val 201) that disrupts the AR/src association is or could be a starting point to reduce the ER-BC spread.

The receptor, however, also influences the growth of HER2+ ER– BC cells in non-genomic way, since in these cells, inhibition of AR by Enzalutamide decreases the HER2 phosphorylation without affecting the total level of HER2 or HER3, and treatment of cells with a combination of enzalutamide and trastuzumab, an approved HER2 target drug, potentiates the inhibitory effect on cell growth due to a single inhibiting drug (49). Giovannelli and coworkers detected that, in TNBC cells, the ligand bound AR rapidly recruits signaling effectors (i.e, Src and PI3-K) linking adhesion and cytoskeleton changes to motility and spreading. Such effect is drastically inhibited by a small peptide disrupting the complexation of AR with Src (unpublished data). These findings offer a good example of non-transcriptional AR action in TNBC. An overview of mechanisms activated by AR in HER+/ER– and TN-BC cells is shown in Figure 3.

In conclusion, it appears that in ER-negative BC cells, AR acts in a more homogeneous way as compared to ER-positive BC cells. In these tumors the receptor clearly promotes cell proliferation and spreading by acting at different levels. This evidence depicts AR as a therapeutic target, potentially very exploitable, for TNBC and provides new opportunities for the treatment of this deadly kind of breast cancer.

## Androgen targeted therapy

Although is not completely clear the influence of circulating androgens in breast cancer development, the role of AR in BC progression seems to be undeniable. In this review we have presented and discussed many findings showing that ligand activation of AR inhibits the cellular proliferation in most of ER-positive BC and induce the cancer growth in most of ER-negative BC. This scenario makes plausible the use of AR modulators or blockers in BCs as shown in Figure 3.

In ER-positive BCs and in a subset of ER-negative BCs in which AR activation inhibits tumor growth, natural and synthetic steroidal androgens (50–53) have been used for therapeutic purpose. Steroidal androgens, however, induce many side effects (54). Thus, recent approaches for the therapy of ER-positive advanced BCs have strongly considered the use of selective AR modulators (SARMs, i.e., enobosarm GTx-024). These compunds activate AR with scant side effects. Enobosarm, for instance, is giving favorable results (55) and is still investigated in a phase II clinical trials in patients with ER+ BC (56).

The most used therapy for advanced BC (Tamoxifen-resistent-BCs and TNBCs) is based on the use of AR antagonists, such as bicalutamide and enzalutamide, a first and second generation AR antagonist, respectively [(41, 42) and TNBC (47)]. Both the antagonists have been used in clinical trials with positive results (57, 58). Other therapies for TNBC are based on the use of CYP17A1 inhibitors, such as abiraterone acetate and seviteronel. These inhibitors reduce the androgen production and the androgen levels. They are now being tested in phase 2 clinical trials (59, 60), alone or in combination with AR antagonists (61).

Preclinical and clinical findings, however, have indicated that AR stimulates the growth of TNBCs or HER2 + BC in combination with other effectors. Some of them are directly involved in cell cycle progression (CDK4/6), some others (PI3-K, Ras, MEK) command the most important intracellular circuits leading to survival, proliferation, invasiveness and drug-escape. Therefore, optimal results might be obtained by approaches in which AR antagonists are used in combination with inhibitors of these patways (4, 62–64).

## Concluding remarks

BC is a heterogeneous disease and, in addition, became frequently resistant to the therapies so far used. Abeit the circulating levels of androgens do not allow a precise understanding of the influence of these hormones in BC development and progression, preclinical and clinical findings have highlighted the role of AR in BC pathogenesis. The concept that AR modulates the growth and progression of BC is currently undeniable. The findings so far reported in literature have also enabled the discovery of new strategies to control this pathology. AR acts in a genomic and in non-genomic way, both in ER-positive and negative BC. It appears, however, that AR can either inhibit or promote the ER+ BC cell growth, while it predominantly stimulates the cellular proliferation and the spreading of ER-BC. AR might act alone or in combination with other effectors participating in intracellular signaling pathways. High-throughput approaches have identified several druggable targets in BC, including the effectors of Src, PI3-K- or Ras-dependent pathways. Currently, targeted agents under investigation include Src, or PI3-K or MEK inhibitors or their combination with hormone therapies or chemotherapeutic agents. Preclinical evidence supports their use in various clinical trials and offers excellent opportunities for the development of new strategies, especially in combination with standard-of-care treatments.

## Author contributions

PG and AM: conceptualization and supervision. PG and MD: writing—original draft. AM, PG, MD, AB, ED, and GG: review and editing.

### Conflict of interest statement

The authors declare that the research was conducted in the absence of any commercial or financial relationships that could be construed as a potential conflict of interest.
